# Extramedullary hematopoiesis in an inguinal lymph node: an unusual presentation of primary myelofibrosis

**DOI:** 10.1186/s12957-022-02660-9

**Published:** 2022-06-08

**Authors:** Nausheen Yaqoob, Neelum Mansoor, Hania Naveed, Saba Jamal

**Affiliations:** 1 Department of Histopathology, Indus Hospital and Health Network, Karachi, 75190 Pakistan; 2 Department of Hematology, Indus Hospital and Health Network, Karachi, 75190 Pakistan

**Keywords:** Primary myelofibrosis, Extramedullary hematopoiesis, Immunohistochemistry, Cytogenetics

## Abstract

**Background:**

Extramedullary hematopoiesis (EMH) is a proliferation of hematopoietic tissue outside of the bone marrow medullary space. It is a pathophysiologic response, more often associated with either a benign reactive hematological disease or a myeloproliferative neoplasm (MPN). Identification of EMH in adults is always pathologic. It is highly unlikely for a myeloproliferative neoplasm to present with inguinal lymphadenopathy. An unusual and complex case can be precisely diagnosed via a multidisciplinary approach involving experts from various modalities of laboratory. In this regard, the present case highlights the importance of an integrated approach in establishing the diagnosis.

**Case presentation:**

We report a case of a 61-year-old male patient of primary myelofibrosis who presented with extramedullary hematopoiesis in an inguinal lymph node. The patient initially presented with generalized symptoms including anemia, fatigue, abdominal pain, and weight loss. On examination, massive splenomegaly. Chest X-ray revealed consolidation which was secondary to right-sided pleural effusion. Therefore, he was suspected to have a lung carcinoma. However, lymph node biopsy revealed extensive fibrosis, consequently effacing the nodal architecture. An abnormal blood picture raised the possibility of bone marrow infiltration. Extensive panel of markers is tested on lymph node and bone trephine. Cytogenetic studies with G-banding analysis and fluorescence in situ hybridization (FISH) played a significant role in deriving clinical decision. Translocations identified in conventional cytogenetic workup led to the diagnosis of primary myelofibrosis. The case is being reported due to unusual presentation of PMF.

**Conclusion:**

In conclusion, it is a distinctive case of myeloproliferative disorder initially presented with extramedullary hematopoiesis and through multidisciplinary workup successfully diagnosed as primary myelofibrosis. Awareness of unique clinical presentations and integrated approach towards diagnosis is the key to such challenging cases.

**Supplementary Information:**

The online version contains supplementary material available at 10.1186/s12957-022-02660-9.

## Background

Extramedullary hematopoiesis (EMH), a pathophysiologic response, is more often associated with either a benign reactive hematological disease or a hematological malignancy. EMH is a proliferation of hematopoietic tissue outside of the bone marrow medullary space. It manifests as a benign proliferation of hematopoietic cells and is most commonly seen in the spleen and liver [1, 2]. Nonetheless, it can also be seen in other organs and patients without a hematologic abnormality [3]. No treatment is generally required for EMH; however, a mass lesion can rarely occur and may require radiation or surgical removal [4].

Though a normal physiologic process in fetal life, EMH has not been identified in adults as physiologic; therefore, it is always considered pathologic [5]. EMH is associated with marrow stromal or bone abnormalities such as Paget’s disease, where there is sufficient bone marrow space for hematopoiesis to occur. It can be associated with benign hematologic disorders, such as thalassemia and other hemoglobinopathies, or malignant bone marrow processes such as myeloproliferative neoplasms [1]. Primary myelofibrosis (PMF) is a chronic myeloproliferative neoplasm that presents with cytopenias, splenomegaly and megakaryocytic proliferation, and atypia in the bone marrow [6]. It is exceptionally unusual to see pleural effusion, lymphadenopathy, and EMH as a presenting clinical feature of PMF.

We present a case of an elderly male who presented with pleural effusion and inguinal lymphadenopathy. It is a unique case of chronic myeloproliferative disorder, presented with EMH and later on diagnosed as PMF on further integrated workup from histopathology, hematology, and cytogenetic departments. The case is being reported to elicit awareness among clinicians and pathologists to proceed with hematological evaluation when an extramedullary site shows the presence of bone marrow elements.

## Case presentation

**Fig. 1:**
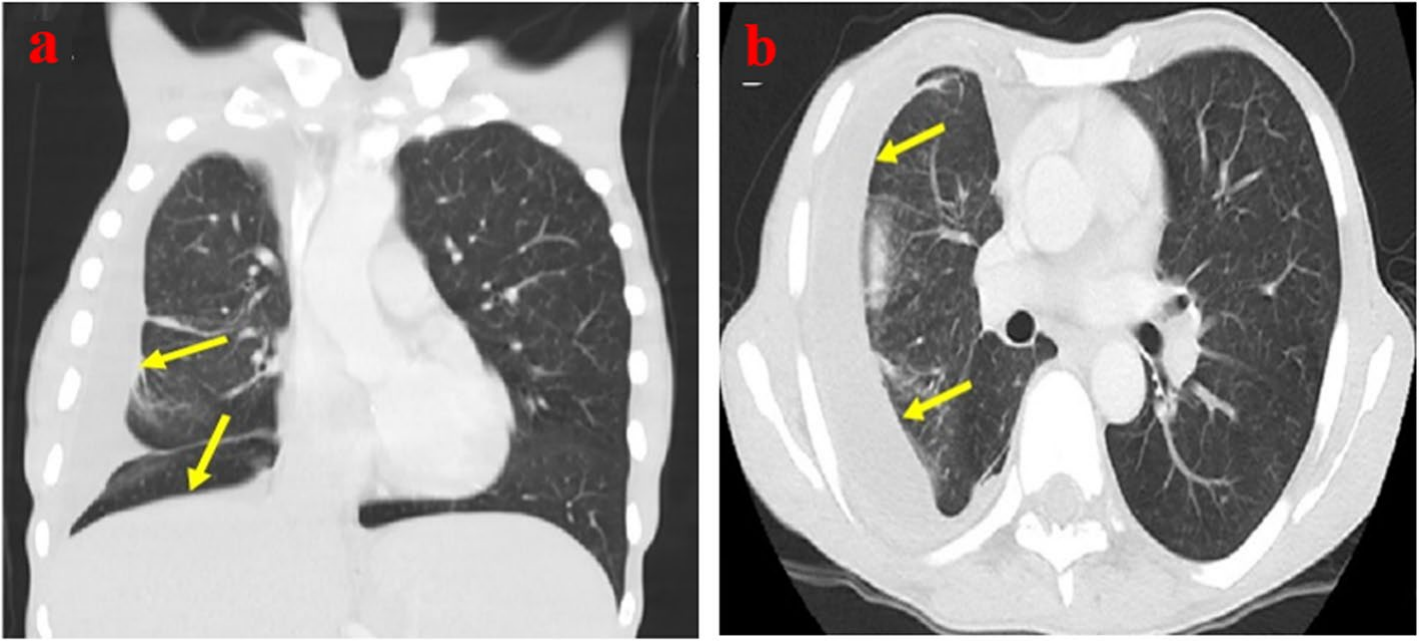
CT scan chest with coronal (**a**) and transverse (**b**) sections of lungs showing right-sided pleural effusion

A 61-year-old male presented with anemia, fatigue, abdominal pain, productive cough, and weight loss for 5 months. Examination showed hepatosplenomegaly and inguinal lymphadenopathy. CT scan chest showed organized right-sided pleural effusion with cicatrization in the upper, middle, and lower right lung (Fig. 1).

**Fig. 2:**
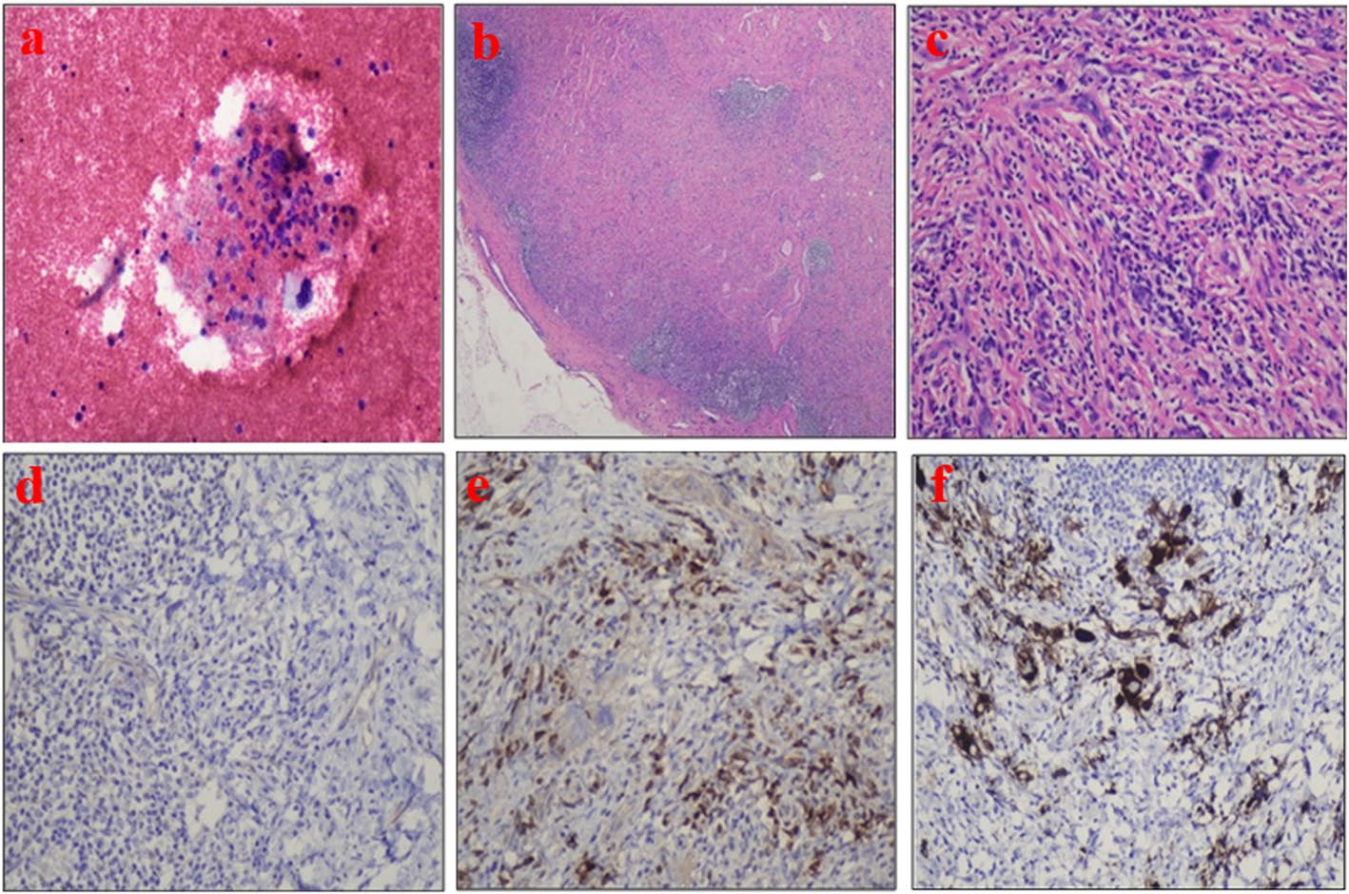
**a** Pleural fluid cytology showing few atypical cells with large hyperchromatic nuclei. **b** H&E showing effacement of the nodal archetecture by extensive fibrosis. **c** H&E showing scattered large atypical cells. **d** CKAE1/AE3 negative. **e** Myeloperoxidase is negative in large atypical cells and highlights background mononuclear cells. **f** CD 61 highlighting atypical cells

Clinical history and radiology raised suspicion of carcinoma lung. Pleural fluid cytology showed few atypical cells (Fig. 2a). The cell block was not prepared due to scanty material; hence, immunohistochemical studies could not be performed, and further characterization was not possible. Therefore, inguinal lymph node biopsy was performed which showed effacement of the nodal architecture by extensive fibrosis (Fig. 2 b–f). Few scattered atypical cells were seen which were negative for CK AE1/AE3, CK 7, CK 20, Ber EP 4, Napsin A, TTF 1, EMA, LCA, CD 30, CD 15, CD 20, CD 3, Tdt, Alk protein, CD 68 (KPI), CD 68 (PGMI), CD 163, and CD 34. After this extensive panel, CD 61 was requested and found to be bright positive in these atypical cells (Fig. 2 e–h). CD117 and MPO highlighted background mononuclear cells of myeloid origin. Hence, the lymph node biopsy was concluded as EMH.

**Fig. 3:**
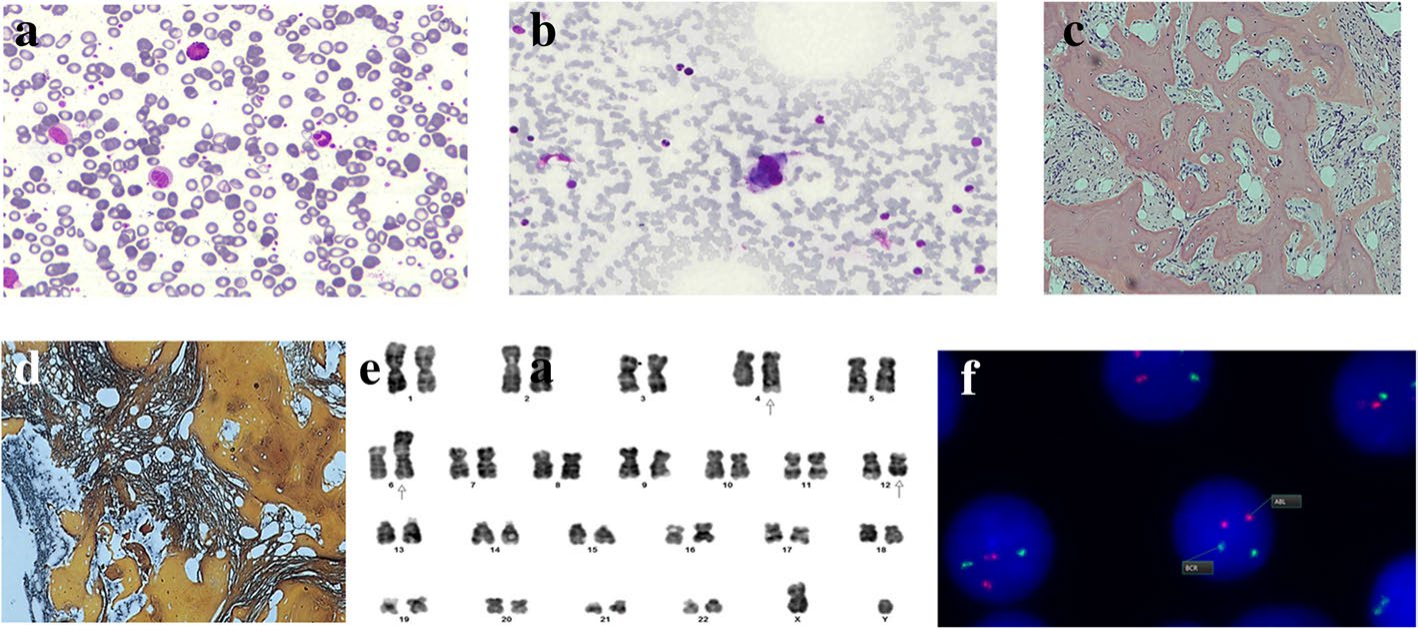
**a** Peripheral blood film exhibits teardrop cells, basophil, and left shift. **b** Bone marrow aspirate showing hypolobated megakaryocyte. **c** H&E stained section of bone marrow trephine biopsy with osteosclerosis and the presence of hematopoietic cells in dilated sinuses. **d** Reticulin stain exhibiting extensive fibrosis. **e** Karyotype. **f** BCR-ABL1 by FISH

Bone marrow biopsy was performed considering abnormal CBC parameters: hemoglobin: 8.6 g/dl, total leukocyte count: 15.3 × 10E9/L, and platelets 206 × 10 E9/L. Peripheral blood film examination revealed anisopoikilocytosis with numerous teardrop cells. Differential count showed neutrophils 37%, lymphocytes 20%, monocytes 12%, eosinophils 0.2%, basophils 0.9%, myelocytes 16%, metamyelocytes 0.2%, and blasts 0.2%. Leukoerythroblastic blood picture raised the possibility of bone marrow infiltration. Bone marrow aspirate was paucispicular; however, all three cell lines were appreciated along with the predominance of myelopoiesis. Bone trephine showed extensive osteosclerotic changes comprising of thickened, broad, and irregular trabeculae occupying > 50% of bone marrow space. New bone formation (osteoid) was also evident at many places, devoid of Howship’s lacunae. There were prominent sinuses consisting of immature hematopoietic precursors, a feature consistent with EMH. Increased megakaryocytes with atypia (anisocytosis, loose clustering, hypolobation, and micromegakaryocytes) were seen (Fig. 3 a–d). An extensive panel of immunohistochemistry is tested that was found to be negative for TdT, CD34, CD3, CD20, CKAE1/AE3, synaptophysin, TTF-1, and PSA; CD45 and MPO showed scattered positivity throughout the sections, while CD61 was increased positive and highlighted megakaryocytic clustering, micromegakaryocytes, and the presence of megakaryocytes within the sinuses. Cytogenetic studies and fluorescence in situ hybridization (FISH) were also performed at the same time (Fig. 3 e–f). FISH for the BCR-ABL1 fusion gene was negative; however, karyotyping using G-banding revealed a balanced translocation between the long arms of chromosomes 4 and 12 and an unbalanced translocation between the long arm of chromosome 1 and the short arm of chromosome 6 resulting in a gain of 1q.

As per the WHO 2017 classification of hematopoietic and lymphoid neoplasms, this specific cytogenetic anomaly is strongly associated with PMF. The diagnosis was further supported by immunohistochemistry where the reactivity to CD61 was in agreement with observed megakaryocytic clusters in bone marrow as well as in lymph node.

## Discussion

The underlying molecular mechanism of EMH in myeloproliferative neoplasms is not known so far, though sequestration and accumulation of clonal myeloid progenitors are suggested to seed hematopoiesis at atypical sites. The resultant EMH can occur in any organ; however, the usual ectopic sites reported so far includes the spleen and liver. However, occasional involvement is reported for the skin, urinary bladder, lymph nodes, etc. [6–8].

Here in this particular case, the clinical history and radiological findings were suggestive of lung carcinoma. Excisional lymph node biopsy when sent for diagnostic or staging workup revealed large abnormal cells in loose clusters along with extensive background fibrosis. These atypical cells upon morphological assessment were identified to be megakaryocytes. An extensive pulmonary workup was done, and no pulmonary pathology was identified in this case. The patient’s chest issues may be related to a widespread hematological disorder that results in the circulation of myeloid progenitors blocking capillaries in the lungs [6]. It is noteworthy to mention here that EMH in a lymph node can be mistakenly diagnosed as metastatic cancer. The megakaryocytes outside the bone marrow may be very dysplastic and can impose a diagnostic challenge in such scenarios. In this regard, immunostaining is quite helpful in setting a differential diagnosis [5]. It is reported in the literature that neoplastic myeloid proliferation at extramedullary sites can be seen in association with myeloproliferative neoplasms, myelodysplastic syndromes, myelodysplastic/myeloproliferative neoplasms, and other myeloid-derived malignancies [9].

Cytogenetic studies played a significant role in deriving clinical decisions in this case. A similar case of PMF along with EMH was reported with a unique manifestation of pleural effusion [8]. EMH associated with PMF usually occurs in the red pulp of the spleen. Reported literature suggests that EMH is a consequence of sequestration and proliferation of circulating clonal myeloid precursors to atypical sites [10]. The translocations observed in the present case are known to be associated with genetic alterations involved in the pathogenesis of PMF. Loss of TP53 plays a critical role in cancer biology and myeloproliferative disorders characterized by wild-type TP53. The major protein regulators of TP53 are MDM2 and MDM4 located on chromosomes 1q and 12q, respectively [11].

## Conclusion

In conclusion, it is a unique case of PMF presented with inguinal lymphadenopathy and pleural effusion. The presence of trilineage hematopoiesis in a lymph node should prompt an immediate search for the underlying hematological disorder. Knowledge and recognition of these cases can help to improve awareness regarding the consideration of a multidisciplinary approach among various sections of the clinical laboratory as a coordinated workup is required in such cases. We aim to report this case to elicit sensitivity that pathologists must consider and correlate all the diagnostic tools including cytogenetic before signing out such ambiguous cases.

## Supplementary Information

**Additional file 1. **Fee waiver letter.

## Data Availability

Available on request

## Abbreviations

CT: Computer tomography
EMH: Extramedullary hematopoiesis
FISH: Fluorescence in situ hybridization
PMF: Primary myelofibrosis
WHO: World Health Organization
