# Supplementary material for: Extramedullary hematopoiesis in an inguinal lymph node: an unusual presentation of primary myelofibrosis
Source: World J Surg Oncol. 2022 Jun 8;20:186. doi: 10.1186/s12957-022-02660-9 (PMC9178870; doi:10.1186/s12957-022-02660-9)
Supplement: Supplementary file 1 — Additional file 1. Fee waiver letter. [file 12957_2022_2660_MOESM1_ESM.pdf]

INDUS HOSPITAL & HEALTH NETWORK

4-7-2022

To
The Chief editor,
World Journal of Surgical Oncology.

Subject: Fee waiver request for the Case Report.

I'm Neelum Mansoor, working as consultant hematologist at The Indus Hospital and Health Network, Karachi, Pakistan request you for a fee waiver for our manuscript case report "Extramedullary Hematopoiesis in an Inguinal Lymph Node; an Unusual Presentation of Primary myelofibrosis" . The case is relevant to the journal and comprised of significant findings pertinent to integrated approach to make a diagnosis where classical clinical and diagnostic features are not present. It is a unique case of primary myelofibrosis presented with extramedullary hematopoiesis that became a diagnostic dilemma.

Indus Hospital & Health Network (IHHN) is a 300-bed tertiary care multidisciplinary non-profit trust based hospital, providing quality care to their patients free of cost. The Indus Hospital Research Center (IHRC) provides a platform for research-related activities at IHHN (clinical laboratory) to guide, support, and stimulate, the scientific research in various disciplines. However, due to financial constraints, we are unable to publish our work in good international journal.

As stated earlier the hospital is mainly governed and supported by philanthropic foundations and donations from general public for its services. Due to limited resources we are not able to afford the publication charges. I would be grateful if you could consider my request to grant us fee waiver for publication charges, for the above mentioned article.

Thank you.

Regards,

Dr. Neelum Mansoor
neelum.mansoor@tih.org.pk
Consultant, Department of Hematology.
The Indus Hospital and Health Network, Karachi, Pakistan.
